# Perceived and Measured Physical Fitness of Police Students

**DOI:** 10.3390/ijerph17207628

**Published:** 2020-10-19

**Authors:** Filip Kukić, Robert G. Lockie, Ana Vesković, Nikola Petrović, Dane Subošić, Danijela Spasić, Darko Paspalj, Lazar Vulin, Nenad Koropanovski

**Affiliations:** 1 Police Sports Education Centre, Abu Dhabi Police, Abu Dhabi 253, UAE; 2 Center for Sport Performance, Department of Kinesiology, California State University, Fullerton, CA 92843, USA; rlockie@fullerton.edu; 3 Faculty of Sport and Physical Education, University of Belgrade, 11000 Belgrade, Serbia; anaveskovic@gmail.com; 4 Department of Psychology, Faculty of Philosophy, University of Belgrade, Čika Ljubina 18-20, 11000 Belgrade, Serbia; nicholas.petrovic@gmail.com; 5 Department of Criminalistics, University of Criminal Investigation and Police Studies, 11080 Belgrade, Serbia; dane.subosic@kpu.edu.rs (D.S.); danijela.spasic@kpu.edu.rs (D.S.); nenad.koropanovski@kpu.edu.rs (N.K.); 6 Faculty of Security Sciences, University of Banja Luka, 78000 Banja Luka, Bosnia and Herzegovina; darko.paspalj@fbn.unibl.org (D.P.); lazar.vulin@fbn.unibl.org (L.V.)

**Keywords:** recruits, tactical fitness, trainee assessment, fitness testing

## Abstract

The physical fitness of police officers needs to support good health and physical performance. Physical fitness comprises a considerable amount of training for police students who are to become police officers. However, to what degree police students are able to perceive their fitness level and differentiate between health-related and performance-related physical fitness is unknown. Therefore, the first aim of this study was to investigate the possibility of differentiation between health-related and performance-related physical fitness using physical self-concept and measured indicators of physical fitness. The second aim of this study was to investigate the association between components of physical self-concept and measured indicators of physical fitness of police students. The sample of 177 police students of both sexes (98 males and 79 females) completed a 40-item physical self-description questionnaire and their physical abilities were assessed for handgrip strength, standing long jump, 30 s sit-ups, and 12-min running. Principal component analysis established health-related and performance-related physical fitness from both perceived and measured physical fitness measures. Correlation analysis revealed a significant relationship between the perceived and measured physical fitness. Results suggest small to moderate ability to recognize the level of certain physical abilities, indicating the association between psychological mechanisms and biological functioning.

## 1. Introduction

Physical fitness has been defined as the ability to complete daily activities without undue fatigue and with enough energy left for pursuing leisure activities [1]. The measurement of physical fitness is an important part of the recruitment and training process of police officers because the job ranges from physically non-demanding (i.e., administrative work) to highly demanding (i.e., chasing, arresting the belligerent or controlling a riot) [2,3,4]. Therefore, next to health-related physical fitness, performance-related physical fitness is also required in certain police occupations [5,6,7]. Accordingly, future police officers (i.e., police students, cadets or trainees) typically complete a physical training program to improve their fitness level before becoming sworn officers [2,4,8]. Furthermore, in some agencies, officers may also complete an annual fitness assessment once they enter the service [9] in order to maintain their physical fitness at the level that provides them with good health and enables them to perform their duties without undue fatigue.

However, the lack of knowledge and resources may result in non-sustainable implementation of strength and conditioning programs across the police agencies and lead to failure to meet the minimal physical fitness standards. Research on exercise within the police agencies showed that strength and conditioning programs are effective only when being controlled, such as during the academy [10,11,12]. However, once police trainees or students enter the police force, their physical fitness typically decreases while their body fat increases as they advance in their career, predominantly due to a lack of planned physical activity and poor nutrition [13,14]. Although socioeconomic, demographic, environmental and cultural factors could have an effect on physical fitness and body composition [15,16,17], the structure and the focus of strength and conditioning programs may be of importance as well [3,10,12].

Typical law enforcement physical fitness programs are often monotonous, and organized in one-size fits all manner [10,11]. Moreover, police students are usually not being taught the basic meaning of exercise or how to conduct the prescribed exercise programs on their own in the absence of an exercise specialist. This could be due to a training schedule that is very dense while at the academy and adding lectures about the basics of implementation of strength and conditioning may pose additional load to the training process. However, strength and conditioning specialists could implement some aspects of teaching police students on the basics of strength and conditioning (i.e., how to apply the prescribed program) through classes already present in the schedule [3]. A convenient way to perform the prescribed programs could be by following the ones written for a certain fitness level that can be assessed by physical test battery or by well-established perceived physical fitness. This may be of importance for continuous work on occupational health and physical performance once students become sworn officers because police agencies do not necessarily provide the access to a strength and conditioning specialist or exercise facility and equipment.

Negative changes in physical activity level, physical fitness level and body composition shortly after cadets complete the training [11,18] could be partially based on whether cadets are competent or motivated to exercise on their own. According to traditional law enforcement academy training, cadets are basically told what to do and how to do it, thereby not being mentally but rather mechanically involved in the process. Therefore, the question arises whether they understand (i.e., perceive) what they were doing and what level they reached (i.e., is their fitness sufficient or insufficient, fair, good, or excellent?) To that end, researchers have developed a multidimensional measurement instrument to assess physical self-concept [19]. Physical self-concept is the individual’s perception of themselves in areas of physical ability and appearance and correlates to measured indicators of physical fitness [19,20,21]. It represents various aspects of the physical self that a person can self-evaluate without actually being assessed for the level of physical abilities that constitute physical fitness. Moreover, studies showed that physical self-concept may increase as the physical fitness improves [22,23]. Lowering the gap between physical self-concept and measured physical fitness measures may be an indicator of the learning process and better understanding of officers’ own fitness status, even though physical fitness objectively increases. This information could be of importance for the sustainable implementation of exercise programs in police occupations because it requires mental engagement (i.e., learning and understanding) as well. Regularly applied, well-planned exercise programs could in return improve occupational health and performance.

However, whether perceived and measured physical fitness of police students differentiate health-related and performance-related indicators of physical fitness and whether perceived and measured physical fitness correlate to each other is unknown. Therefore, the first aim of this study was to investigate the possibility of differentiation between health-related and performance-related physical fitness using physical self-concept and measured indicators of physical fitness. The second aim of this study was to investigate the association between components of physical self-concept and measured indicators of physical fitness of police students. The first hypothesis was that health-related and performance-related physical fitness would differentiate whether assessed by self-concept or measured by physical fitness tests. The second hypothesis was that components of physical self-concept would be associated with corresponding measured components of physical fitness.

## 2. Materials and Methods

### 2.1. Participants

The sample consisted of 177 police students of both sexes (98 males and 79 females) from the University of Criminal Investigation and Police Studies, Serbia. The main characteristics of males were age = 20.6 ± 1.3 years, body height = 183.26 ± 6.46 cm, body weight = 82.6 ± 9.2 kg, and body mass index (BMI) = 24.6 ± 1.9 kg/m^2^. The main characteristics of females were age = 20.9 ± 1.4 years, body height = 170.6 ± 4.6 cm, body weight = 63.9 ± 6.4 kg, and BMI = 21.7 ± 1.7 kg/m^2^. Considering that all students had to go through the vigorous selection process to be recruited including health screening, psychological assessment, intellectual properties and physical fitness, the sample could be considered healthy and relatively homogenous by physical fitness. All participants signed the terms of fee and informed consent. The procedure was conducted with the permission of the Ethics Committee of the University of Criminal Investigation and Police studies, Serbia (440-2) and was performed in accordance with the Helsinki Declaration.

### 2.2. Procedures

A cross-sectional research design was applied to investigate the distinguishing properties of physical self-concept and measured components of physical fitness. The physical self-description questionnaire (PSDQ) was administered via the Moodle platform, which students could access through their university profile two weeks before the end of the academic year 2019/2020. Participants were asked to report their age and gender, and whether they were recently unable to be physically active due to illness or injury. No participant reported issues in being physically active.

Measured components of physical fitness were assessed one week after the PSDQ and included hand grip strength for maximal muscular force, standing long jump for lower body muscular power, 30-sec push up test for local muscular endurance, 12-min Cooper running test for aerobic endurance and BMI for nutritional status. Hand grip strength indicates potential for muscle contraction (i.e., muscle quality) [24,25], while BMI indicates the ratio between body mass and body height, which can range from underweight to over normal weight to overweight [1]. Although increased body mass relative overall body size (i.e., height^2^), this may occur due to an increase in skeletal muscle mass [26,27], more often in police officers it suggests accumulation of excess fat mass [28,29]. In contrast, body mass that is too low relative to the size of the individual may suggest insufficient muscle mass [30]. Therefore, these two indicators together provide some measure of the general health status and could be considered health-related components of physical fitness [1,31]. Standing long jump, 30-sec push up test and the 12-min Cooper running test were the tests of physical performance and could be considered as performance-related physical fitness [6].

### 2.3. Physical Self-Concept

Multi-dimensional construct of physical self-concept was assessed using a 40-item physical self-description questionnaire (PSDQ-S) [19]. The PSDQ-S was designed to measure global physical self-concept and global esteem as well as nine components that relate to physical self-concept. These include physical activity, appearance, body fat, coordination, endurance, flexibility, health, sport competence and strength. The components were explained according to Marsh and Redmayne [21] and are as follows:

- Global physical self-concept—feelings about one’s physical self.
- Self-esteem—overall positive feelings about oneself.
- Physical activity—being physically active, completing physical activity regularly.
- Appearance—attractiveness.
- Body fat—not being overweight, not being too fat.
- Coordination—being good at coordinated movements, doing physical movements smoothly.
- Endurance—the ability to run a long distance without stopping, not getting tired too easily when exercising hard.
- Flexibility—the ability to bend and turn your body easily in different directions.
- Health—not being sick, recovering quickly when sick.
- Sport competence—being good at sport, being athletic, having good sports skills.
- Strength—being strong, great musculature.

Each PSDQ item is a simple declarative statement about physical appearance and abilities. The individuals respond using a 6-point Likert scale (1-completely false; 2-mostly false; 3-more false than true; 4-more true than false; 5-mostly true; 6-completely true) reporting the extent to which they endorsed statements. A long form of the instrument was previously translated and used in Serbia and has demonstrated acceptable psychometric qualities [32,33]. In our study, Cronbach’s alpha for the subscale’s health, physical activity and body fat ranged from 0.601 to 0.665 and from 0.749 to 0.940 for remaining subscales.

### 2.4. Physical Abilities

*Handgrip strength*—A standardized hand grip test with a tensiometric probe was used for the measurement of maximal isometric force of dominant finger flexors following a previously reported procedure [34,35]. In short, participants were standing upright holding the measuring device alongside the body. The hand holding the device with the probe was approximately 10 cm away from the trunk. The participants were not allowed to move from the initial position during the test trial. The measurement was performed using Sports Medical Solutions Handgrip system (SMS HG, Belgrade, Serbia), with precision of the probe set at the level of ±0.01 N. The system measuring chain consisted of a load cell, an acquisition unit with integrated 12-bit A/D conversion and signal conditioning, and a laptop with installed SMS proprietary software. The sampling frequency of the system was 500 Hz. Sensor calibration was performed using laboratory weights. Reliability of the SMS device was recently investigated elsewhere, showing high reliability (ICC = 0.948) [35].

*Standing long jump*—Lower-body power in the horizontal plane was assessed by a standing long jump (SLJ) test following the procedures of Pihlainen et al. [36]. The participants were instructed to jump as far as possible from the marked line with both feet, with no restrictions placed on the degree of arm swing or countermovement used. The distance from the starting to the landing point at the heel contact was measured in centimeters with 1 cm measurement precision.

*30s Sit Ups*—The endurance of the abdominal and hip flexor muscles was assessed using the sit-up (SU) test with their hands behind the head, wherein participants completed as many repetitions as possible within 30 s [2,37]. The participants were instructed to touch the floor with both shoulder blades every time and not to raise their hips from the floor at any time.

*12min Run*—General aerobic endurance was assessed using the 12-min Cooper running test (RUN), whereby the participants were required to cover the longest possible distance in 12 min. This was shown to be a valid test (r = 0.93, *p* < 0.001) estimator of maximal oxygen consumption [38]. The participants ran around the 400 m long circuit track marked at every five meters for the required time.

### 2.5. Statistics

The descriptive statistics were calculated for mean and standard deviation for the whole sample, as well as separately for males and females. Principal component analysis was used to extract the main components of self-described and measured physical fitness, which then were labelled according to the structure of the extracted components. The Kolmogorov–Smirnov test of normality of data distribution was applied. Spearman correlation analysis was used as the data distribution was not normal for the self-concept components. The strength of the correlations were rated as small = 0.20–0.49, medium = 0.50–0.79, and large ≥ 0.8 [39]. Statistical procedures were conducted using Statistical Package for Social Sciences (SPSS, 20.0, IBM, Chicago, IL, USA).

## 3. Results

The descriptive statistics for the mean, standard deviation, and Kolmogorov–Smirnov test of data distribution is shown in Table 1. In total, 8 out of 11 physical self-concept variables had non-normal distribution in males, and 5 out of 11 in females. The data distribution was normal in all measured physical fitness indicators in males, while one out of four variables was not normally distributed in females. Thirty male students had BMI over 25.0 kg/m^2^, with eight being above 27.5 kg/m^2^, while only four female students had BMI over 25.0 kg/m^2^, with one being over 27.5 kg/m^2^. None of the participants had BMI over 30 kg/m^2^.

PCA extracted two main components of perceived physical fitness for the whole sample as well as for males and females (Table 2). Considering the structure of the components, component 1 could be labelled as performance-related perceived fitness and component 2 could be labelled health-related perceived fitness. PCA extracted one main component of perceived physical fitness for the whole sample and two main components for males and females (Table 3). Considering the structure of the components, component 1 could be labelled as performance-related physical-fitness and component 2 could be labelled health-related physical fitness.

Significant correlations occurred between several components of physical self-concept and measured physical fitness (Table 4). Considering the whole sample, only Health, Appearance and Flexibility did not correlate to any measured fitness indicator. Considering the whole sample, perceived strength and endurance correlated with HGS, SLJ, SU, RUN and BMI. However, when analyzed by sex, perceived strength correlated only to HGS and BMI in males and SU in females, while perceived endurance correlated to RUN performance in both sexes. The highest correlation between perceived and measured indicators occurred between body fatness and BMI in general as well as in both sexes.

## 4. Discussion

This study firstly investigated the possibility of differentiating between health-related and performance-related physical fitness using perceived and measured indicators of physical fitness in police students. Secondly, this study detailed relationships between perceived and measured indicators of physical fitness. The results suggested that police students’ physical fitness could be classified as health-related and performance-related whether self-reported on their physical abilities or if their physical abilities were assessed. In addition, significant correlations occurred between the measured and perceived body fatness, strength and endurance. Therefore, both hypotheses were true.

PCA showed that a similar amount of variance was explained by PSDQ-S and measured indicators of fitness. Moreover, components of PCA obtained from these two explained similar amounts of variance in health-related and performance-related components of physical fitness. Therefore, it seems that subjective and objective measures of physical fitness tend to group similarly, providing the evidence for the theoretical approach to physical fitness as being divided into health-related and performance-related. Considering this, having good measured levels at both aspects of physical fitness and being accurate in self-evaluating them may indicate a certain level of conscious approach to physical health.

These results seem to be nested in the combination of human biological functioning and permanent conscious and unconscious anxiety for health and the desire to maintain health, as well as in perceiving the importance of physical performance and the actual need for it [40,41,42]. Although significant relationships between perceived and measured physical fitness may support this notion, the coefficients were small. These data indicated that the students’ ability to recognize the level of physical fitness and its certain components was low. The strongest associations occurred between self-perceived body fatness and BMI. This was followed by the associations between perceived and measured endurance and then strength. Considering this, it seems that the accuracy of self-perception is higher when students are required to self-evaluate fitness components that they are more familiar with, which is in accordance with results from prior research [20,43]. Therefore, familiarizing police students with wider range of physical abilities through education may improve the accuracy of their physical self-concept.

Physical fitness is fundamentally important for the physical performance and health of police students and officers [7,44]. Police students with higher level of physical fitness are less likely to get injured and drop from the academy [4], and select fitness measures (e.g., sit-ups and aerobic running performance) have been positively associated with grade point average and faster graduation [2]. Furthermore, fitter police officers tend to perform better in occupational tasks [45,46]. In contrast, police officers with increased BMI over the criterion (i.e., over 30 kg/m^2^) level possess a higher health risk [47,48]. Therefore, being aware of the physical self and accurately perceiving physical fitness could be an advantage for those engaged in policing as officers could react in a more timely manner if negative trends occur in physical fitness [49,50]. Improving physical fitness while at the academy and being able to maintain fitness while being a sworn officer, should positively impact an officer’s health and physical performance. In return, this could save resources that police agencies spend on training and health insurance, as unfit officers and/or with higher adiposity levels may be more prone to injuries and other health-related absenteeism [51,52].

### Limitations

A longitudinal design should be applied to investigate if the physical self-concept changes after the exercise or education intervention. Body fat level, flexibility, coordination, sports competency and the level of physical activity should be directly measured and compared against self-reported data to confirm the accuracy of this information in police populations. The sample could be extended on students from other faculties and across different age categories. Moreover, police officers should be assessed cross-sectionally and investigated in longitudinal experimental design so the concept of self-reporting physical fitness could be defined.

## 5. Conclusions

This is the first study that investigated physical self-concept and measured physical fitness in this specific population. The obtained results provide support for the association between the perceived and measured physical fitness of police students. Students perceived two types of physical fitness that had been well established in literature on physical fitness: health-related and performance-related. This suggests that the psychological, background mechanisms follow biological functioning of the body. Significant correlations between measured and perceived fitness measures indicated that students may be able to recognize the level of certain physical abilities. Physical self-concept could be used occasionally in a practical setting as it could provide an insight into one’s physical fitness status and indirectly the history of physical activity and exercise. Moreover, improved precision of physical self-concept may increase the awareness of the physical self and the objectivity of perception of physical performance. This could be of importance for exercise behavior as police students could apply more precise strength and conditioning programs in their leisure time that would aim to prove certain components of fitness (i.e., those perceived as insufficient). Considering this, future research on this population should investigate whether longitudinal exercise intervention and education could increase students’ ability to self-evaluate physical abilities.

## Figures and Tables

**Table 1:** Descriptive statistics for male and female police students.

Variables	Whole Sample (N = 177)			Males (n = 98)			Females (n = 79)		
	Mean	SD	KST	Mean	SD	KST	Mean	SD	KST
Global self-concept	5.31	0.78	0.000	5.39	0.73	0.00	5.20	0.84	0.02
Self esteem	4.54	0.55	0.008	4.61	0.53	0.03	4.46	0.58	0.37
Health	3.76	0.37	0.000	3.73	0.42	0.00	3.80	0.29	0.00
Coordination	5.18	0.75	0.003	5.28	0.70	0.01	5.05	0.80	0.21
Physical activity	4.10	0.99	0.049	4.26	0.89	0.14	3.89	1.06	0.54
Body fatness	3.45	0.69	0.000	3.44	0.69	0.01	3.47	0.69	0.00
Sport competency	4.85	0.98	0.004	5.00	0.91	0.02	4.65	1.03	0.37
Appearance	4.79	0.85	0.015	4.84	0.78	0.15	4.73	0.93	0.19
Strength	5.00	0.84	0.001	5.22	0.76	0.02	4.72	0.86	0.07
Flexibility	4.56	1.06	0.002	4.52	1.02	0.11	4.62	1.12	0.03
Endurance	4.47	1.07	0.047	4.70	1.03	0.06	4.19	1.06	0.27
Hand grip strength (daN)	52.41	13.49	0.002	63.19	7.24	0.23	39.03	4.26	0.70
Standing long jump (cm)	210.45	29.80	0.006	233.32	15.98	0.28	182.08	14.63	0.71
30 s Sit ups (No)	24.76	2.91	0.088	26.18	2.71	0.22	22.99	2.05	0.03
12 min Run (m)	2480.17	334.13	0.002	2731.43	171.89	0.09	2168.48	193.52	0.53
BMI (kg/m^2^)	23.40	2.26	0.836	24.55	1.95	0.41	21.96	1.74	0.95

SD—standard deviation, KST—Kolmogorov–Smirnov test.

**Table 2:** Principal component analysis for perceived components of physical fitness.

Whole Sample			Males			Females		
Variables	Component (59.0%)		Variables	Component (61.3%)		Variables	Component (58.4%)	
	1 (47.6%)	2 (11.4%)		1 (49.6%)	2 (11.7%)		1 (45.4%)	2 (12.9%)
Global self-concept	0.889		Global self-concept	0.895		Global self-concept	0.890	
Self esteem	0.840		Strength	0.831		Self esteem	0.851	
Coordination	0.804		Self esteem	0.812		Coordination	0.804	
Strength	0.797		Endurance	0.793		Strength	0.748	
Endurance	0.733		Coordination	0.781		Flexibility	0.735	
Sports competency	0.689		Sports competency	0.726		Physical appearance	0.681	
Physical activity	0.688		Physical activity	0.705		Physical activity	0.660	
Appearance	0.680		Physical appearance	0.688		Endurance	0.642	
Flexibility	0.674		Flexibility	0.654		Sports competency	0.626	
Body fatness		0.781	Body fatness		0.746	Body fatness		0.854
Health		0.752	Health		0.704	Health		0.720

**Table 3:** Principal component analysis for measured physical fitness components.

Whole Sample		Males			Females		
Variables	Component (69.0%)	Variables	Component (62.6%)		Variables	Component (57.8%)	
	1		1 (37.6%)	2 (25.1%)		1 (33.3%)	2 (24.6%)
SLJ	0.930	SLJ	0.793		SLJ	**0.762**	
HGS	0.928	SU	0.763		RUN	0.673	
RUN	0.883	RUN	0.650		SU	0.561	
SU	0.709	BMI		0.834	BMI		0.794
BMI	0.667	HGS		0.778	HGS		0.787

SLJ—Standing long jump, HGS—Handgrip strength, RUN—12-min Cooper running test, SU—30 s Sit up test, BMI—Body mass index.

**Table 4:** Correlation analysis for the whole sample, male and female police students.

Variables	HGS			SLJ			SU			RUN			BMI		
	WS	M	F	WS	M	F	WS	M	F	WS	M	F	WS	M	F
Global self-concept	0.13	0.13	−0.16	0.18 ^†^	0.12	0.17	0.28 ^††^	0.11	0.26 ^†^	0.19 ^†^	0.14	0.13	0.14	0.20 ^†^	0.04
Self esteem	0.15	0.09	−0.12	0.17 ^†^	0.13	0.02	0.18 ^†^	0.04	0.21	0.18 ^†^	0.14	0.01	0.23 ^†^	0.26 ^†^	0.17
Health	−0.03	0.04	0.03	0.00	0.08	0.05	0.05	0.04	0.13	−0.02	0.11	0.08	−0.01	0.06	−0.07
Coordination	0.13	0.01	−0.04	0.15	0.06	0.06	0.19 ^††^	−0.01	0.24 ^†^	0.14	−0.02	−0.01	0.12	0.06	0.04
Physical activity	0.12	0.03	−0.19	0.23 ^††^	0.13	0.17	0.24 ^††^	0.09	0.25 ^†^	0.19 ^††^	0.02	0.17	0.18 ^†^	0.19	0.09
Body fatness	0.07	0.14	0.08	−0.07	−0.23 ^†^	−0.06	−0.11	−0.21 ^†^	−0.07	−0.05	−0.18	−0.09	0.43 ^††^	0.43 ^††^	0.50 ^††^
Sport competency	0.17 ^†^	0.10	0.01	0.21 ^††^	0.12	0.12	0.30 ^††^	0.17	0.25 ^†^	0.19 ^†^	0.16	−0.16	0.10	0.12	−0.11
Appearance	0.05	0.12	−0.10	0.07	0.08	0.08	0.13	0.10	0.15	0.04	−0.07	−0.05	−0.01	0.06	−0.08
Strength	0.35 ^††^	0.25 ^†^	0.07	0.30 ^††^	0.14	0.04	0.30 ^††^	0.13	0.17	0.31 ^††^	0.09	0.13	0.34 ^††^	0.20 ^†^	0.27 ^†^
Flexibility	−0.09	−0.07	−0.12	−0.00	0.15	0.04	0.07	0.12	0.11	−0.06	−0.03	−0.09	−0.03	−0.08	0.16
Endurance	0.20 ^††^	−0.00	−0.03	0.23 ^††^	0.05	0.05	0.23 ^††^	0.06	0.14	0.36 ^††^	0.25 ^†^	0.26 ^†^	0.19 ^††^	0.11	0.05

^††^ Spearman’s correlation significant at *p* < 0.01, ^†^ Spearman’s correlation significant at *p* < 0.05. HGS—Handgrip strength, SLJ—Standing long jump, SU—30-sec Sit ups, RUN—12-min Running test, BMI—Body mass index. WS—Whole sample, M—Males, F—Females.
